# Supplementary material for: Mitochondrial complexome reveals quality-control pathways of protein import
Source: Nature. 2023 Jan 25;614(7946):153–9. doi: 10.1038/s41586-022-05641-w (PMC9892010; doi:10.1038/s41586-022-05641-w)
Supplement: Supplementary file 1 — Supplementary Fig. 1 and Supplementary Tables 4–6. [file 41586_2022_5641_MOESM1_ESM.pdf]

nature portfolio

https://doi.org/10.1038/s41586-022-05641-w

Supplementary information

Mitochondrial complexome reveals quality-control pathways of protein import

In the format provided by the authors and unedited

Supplementary Information Guide

Mitochondrial complexome reveals quality control pathways of protein import

Uwe Schulte et al.

Supplementary Figure 1. Uncropped versions of gels/blots of this study.

Supplementary Table 1. MS data related to identification/quantification of mitochondrial proteins (separate Excel file). Sheet 1: MitCOM proteins (total of 906 proteins); sheet 2: mitochondrial proteins with single-peptide detection (total of 49 proteins).

Supplementary Table 2. Data underlying abundance-mass profiles of MitCOM proteins (separate Excel file).

Supplementary Table 3. MS data related to identification/quantification of non-mitochondrial proteins (total of 985 proteins) **(separate Excel file).**

Supplementary Table 4. Yeast strains used in this study.

Supplementary Table 5. Plasmids used in this study.

Supplementary Table 6. Antibodies used in this study.

Supplementary Figure 1. Uncropped versions of gels/blots of this study.

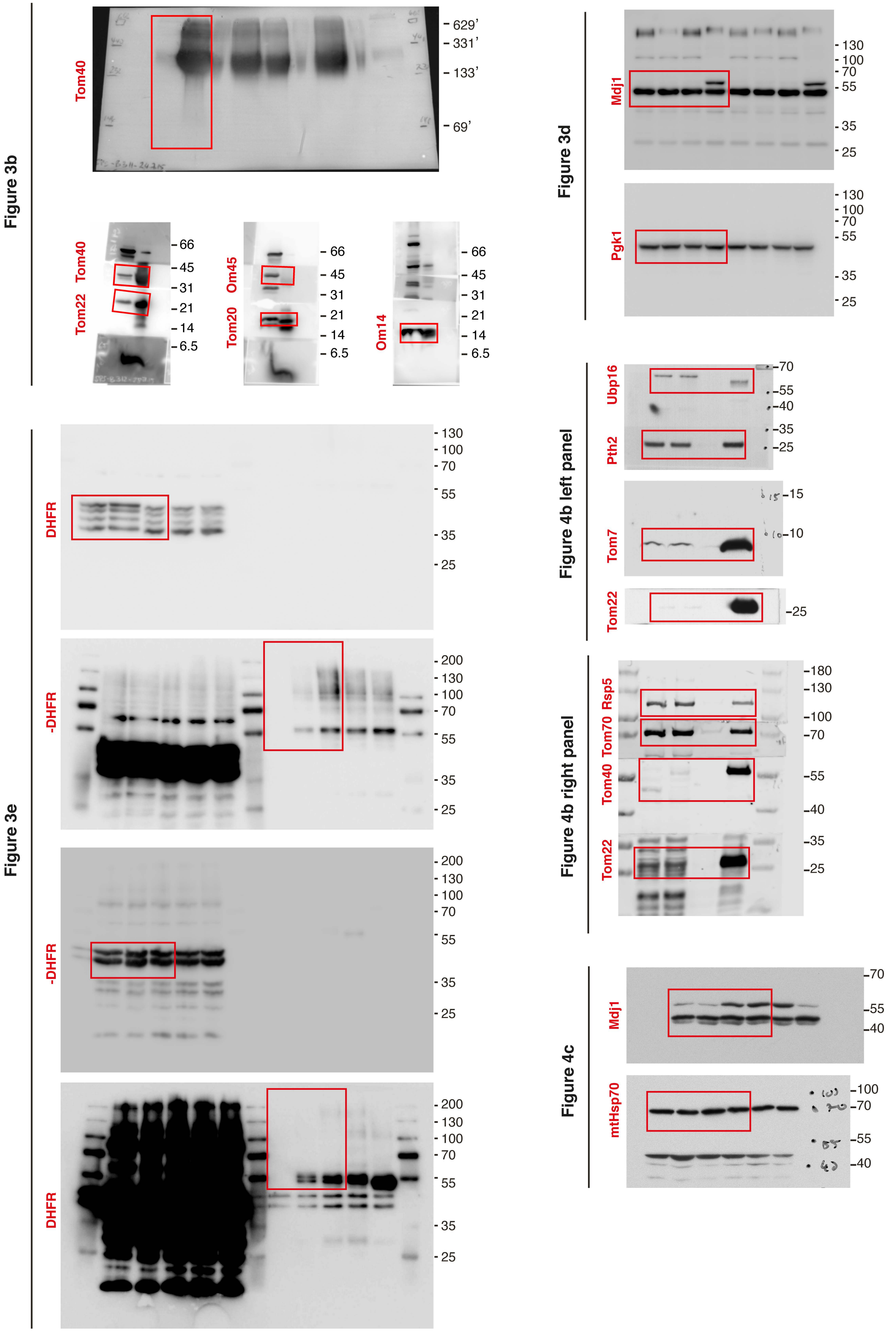

Figure 3b
Tom40
629'
331'
133'
69'
Tom40
Tom22
Om45
Tom20
Om14
66
45
31
21
14
6.5
Figure 3e
DHFR
-DHFR
-DHFR
DHFR
200
130
100
70
55
35
25
Figure 3d
Mdj1
Pgk1
130
100
70
55
35
25
Figure 4b left panel
Ubp16
Pth2
Tom7
Tom22
70
55
40
35
25
15
10
Figure 4b right panel
Rsp5
Tom70
Tom40
Tom22
180
130
100
70
55
40
35
25
Figure 4c
Mdj1
mtHsp70
100
70
55
40

Figure 4d

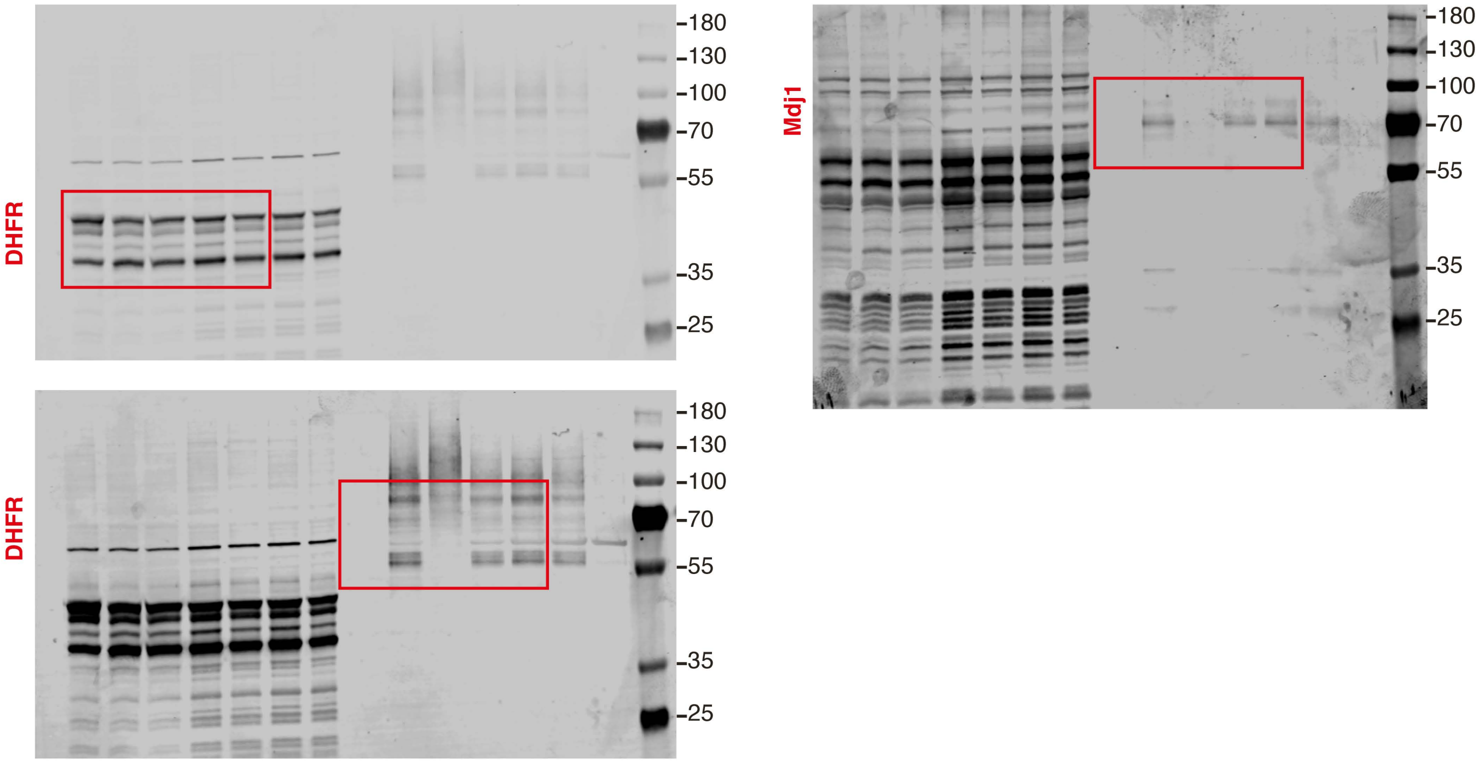

Figure 4e

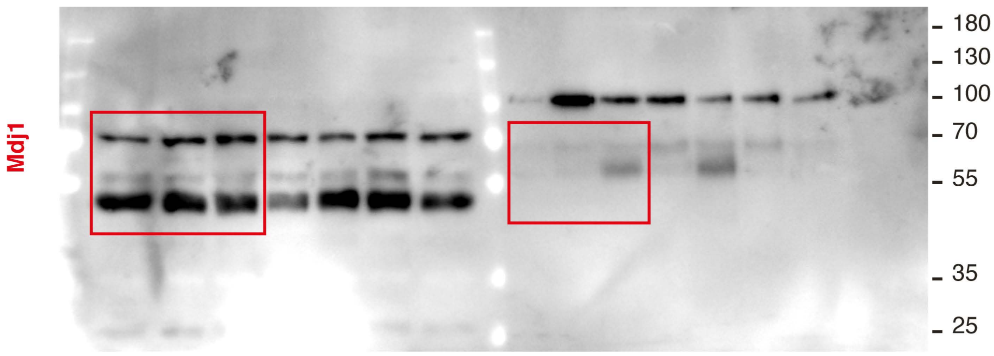

Figure 4f

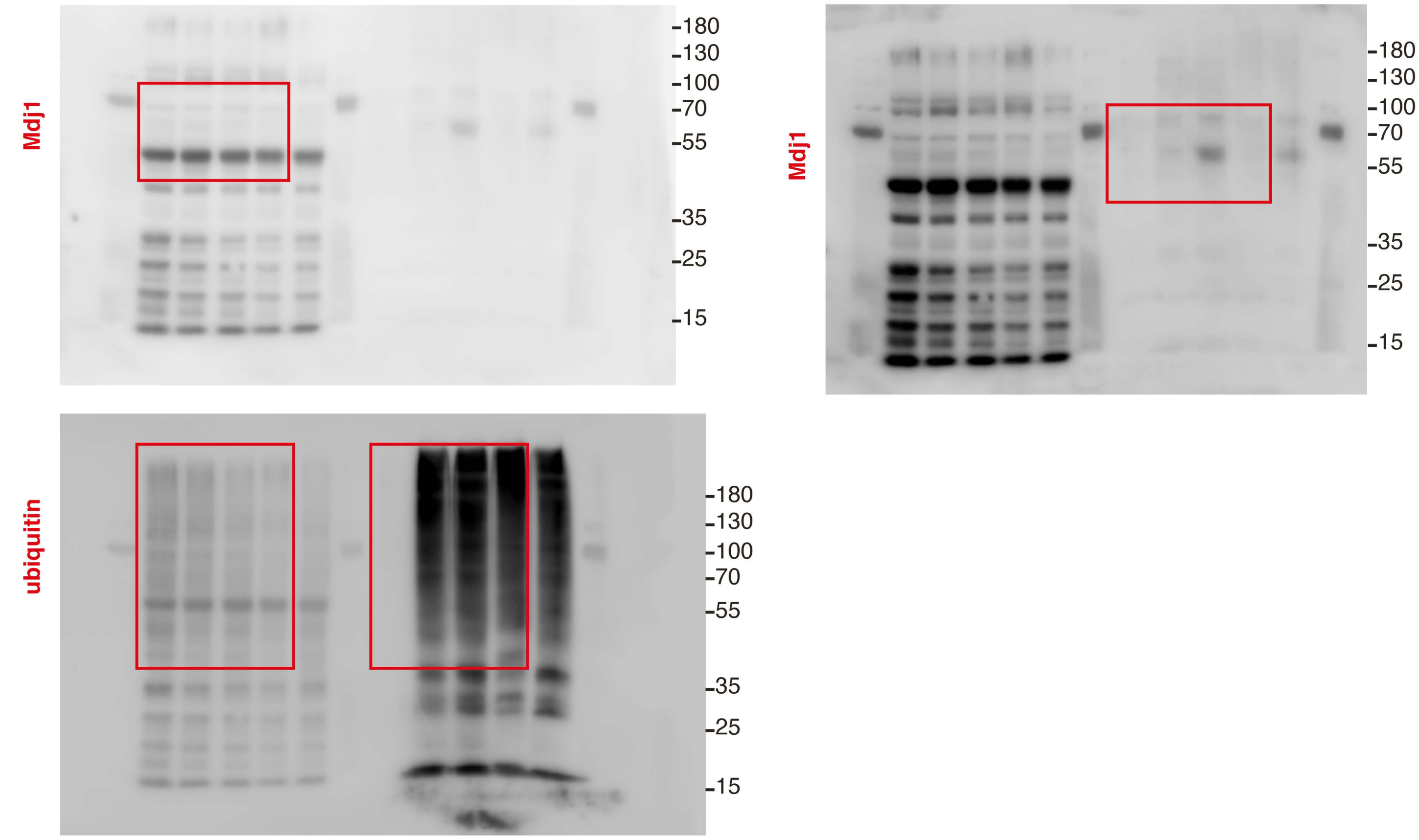

Figure 4g

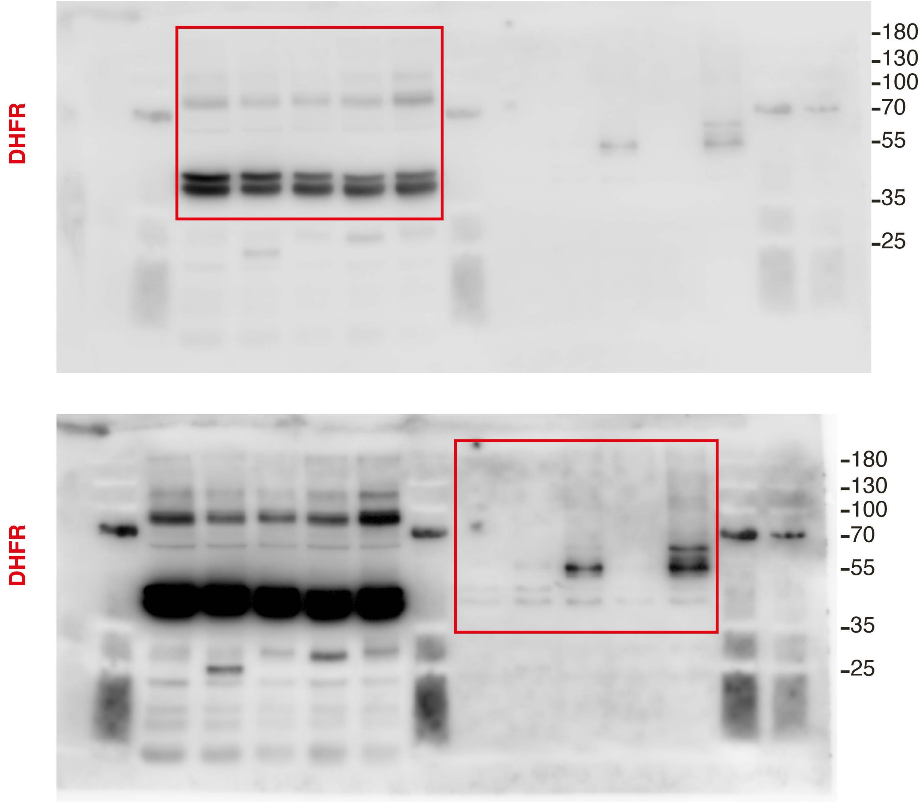

Figure 5a

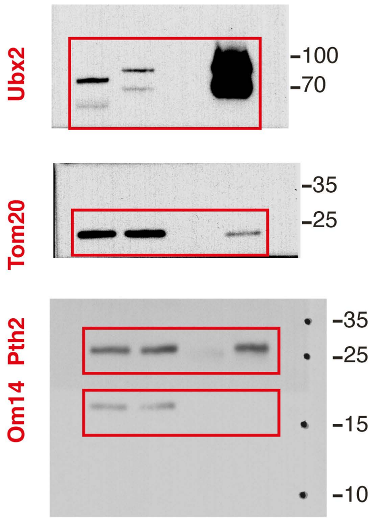

Figure 5b

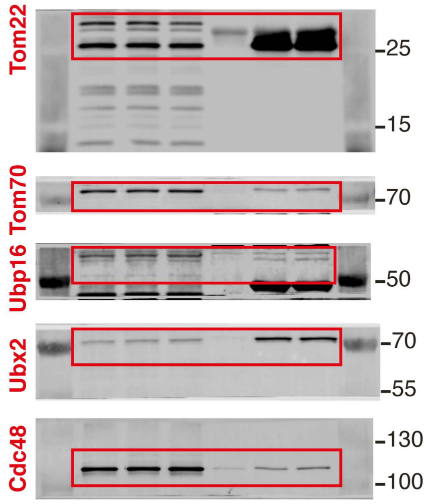

Figure 5c

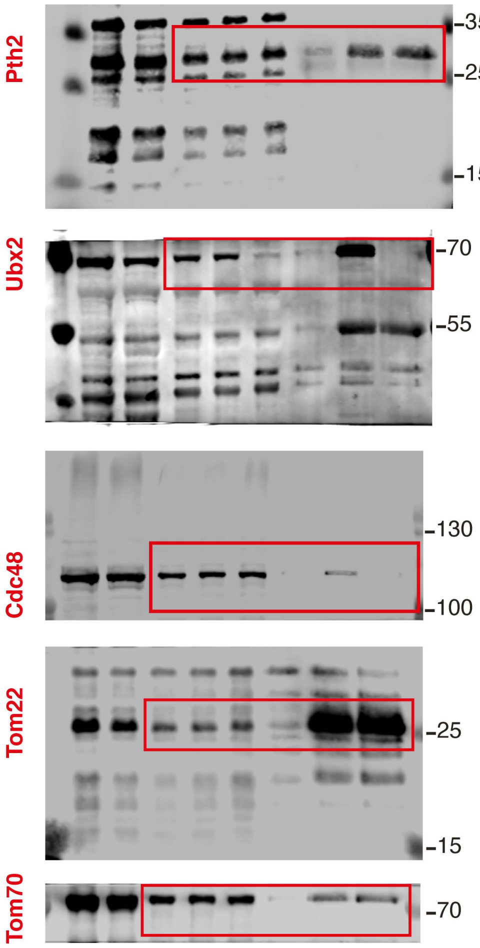

Figure 5e

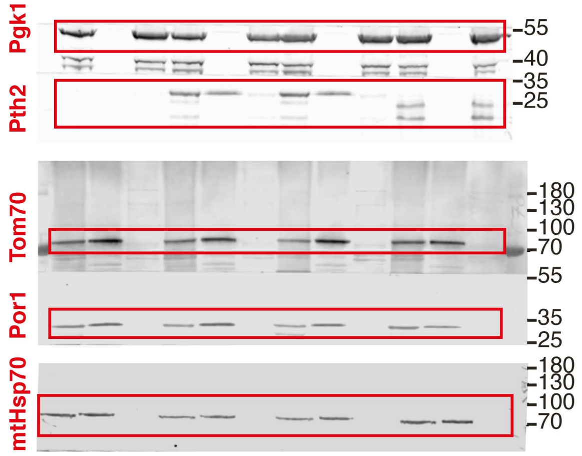

Figure 5g

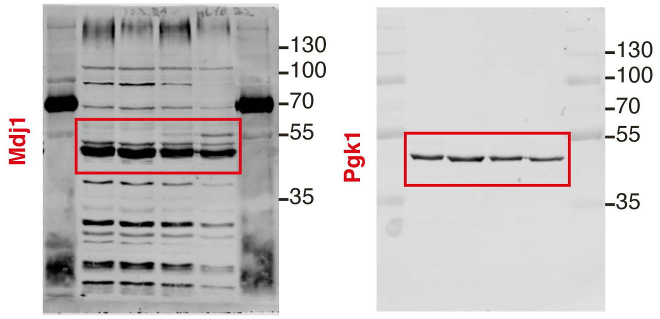

Extended Data Figure 8f

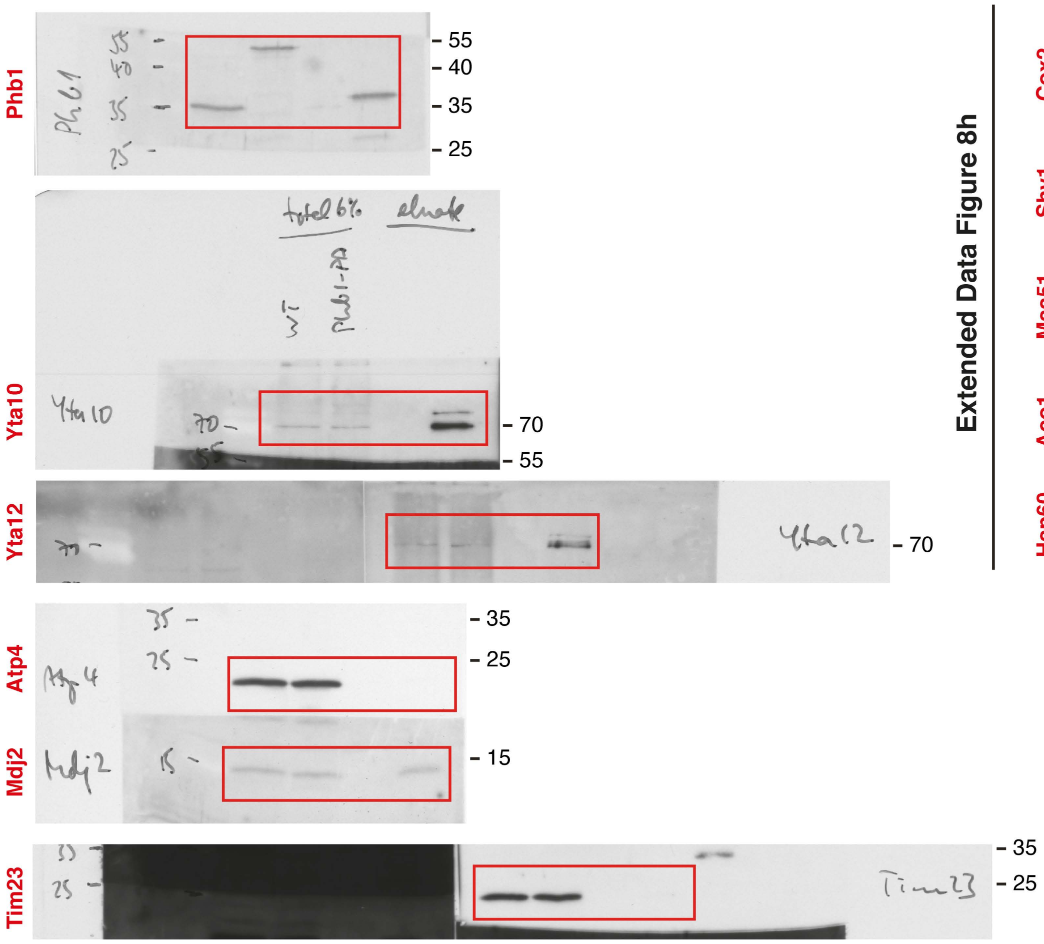

Extended Data Figure 8h

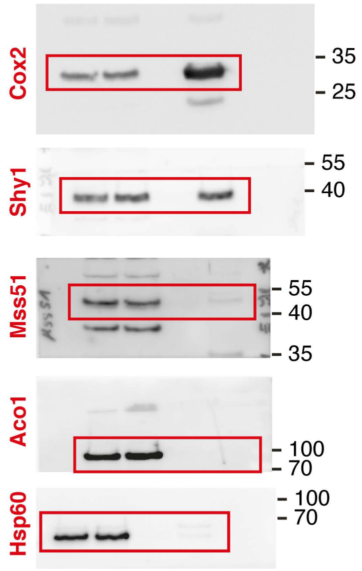

Extended Data Figure 9b

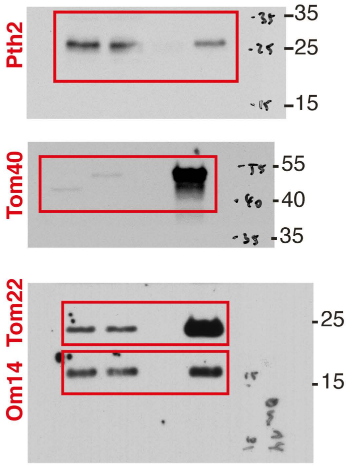

Extended Data Figure 9c

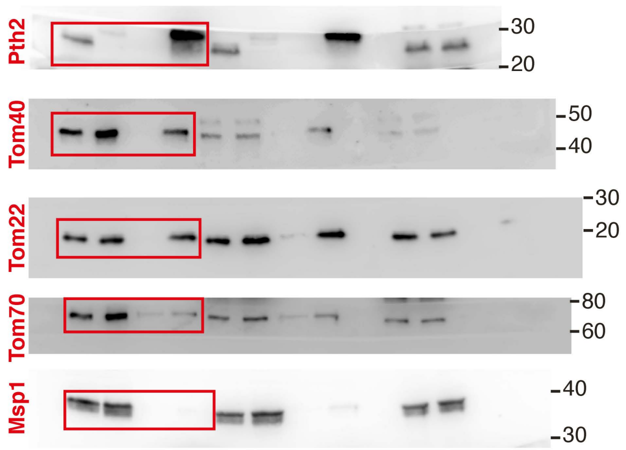

Extended Data Figure 9f

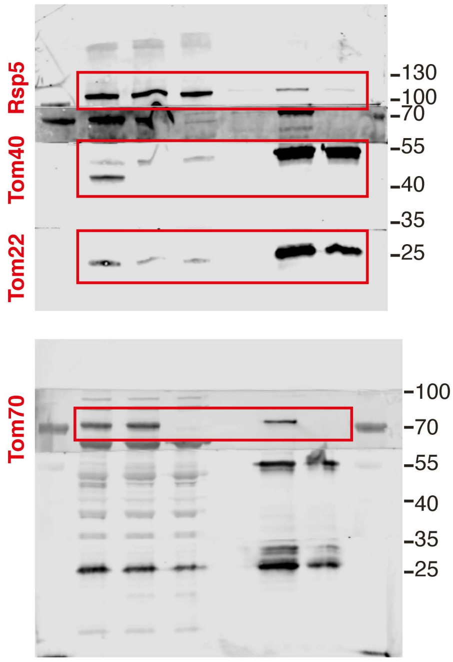

Extended Data Figure 9g

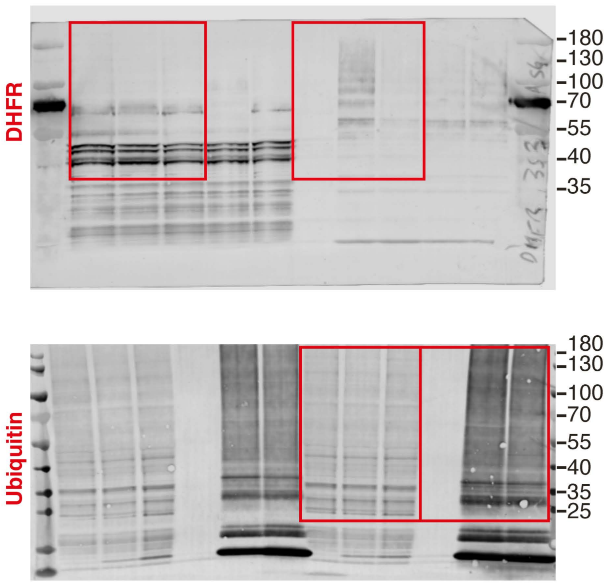

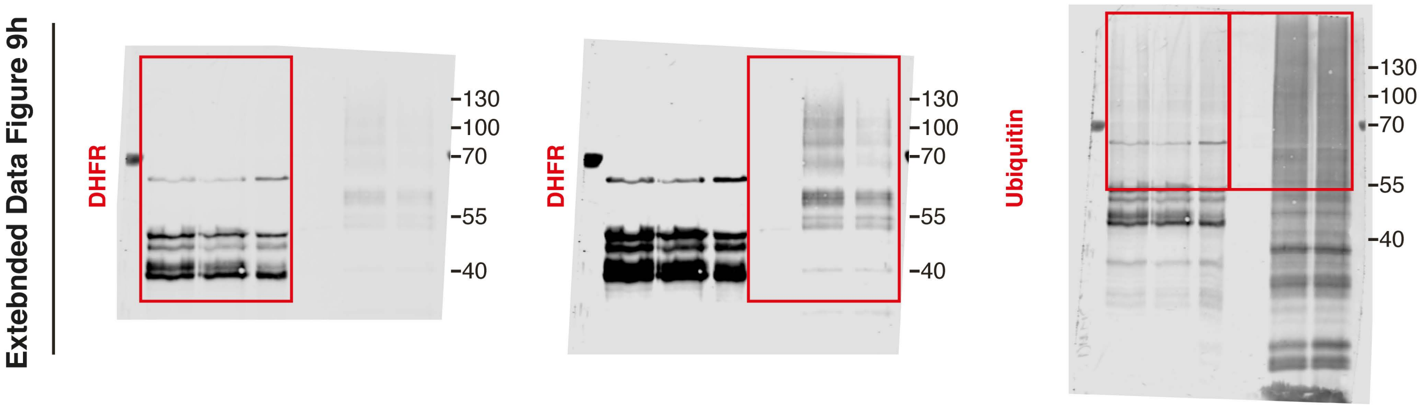
Extebnded Data Figure 9h
DHFR
130
100
70
55
40
DHFR
130
100
70
55
40
Ubiquitin
130
100
70
55
40

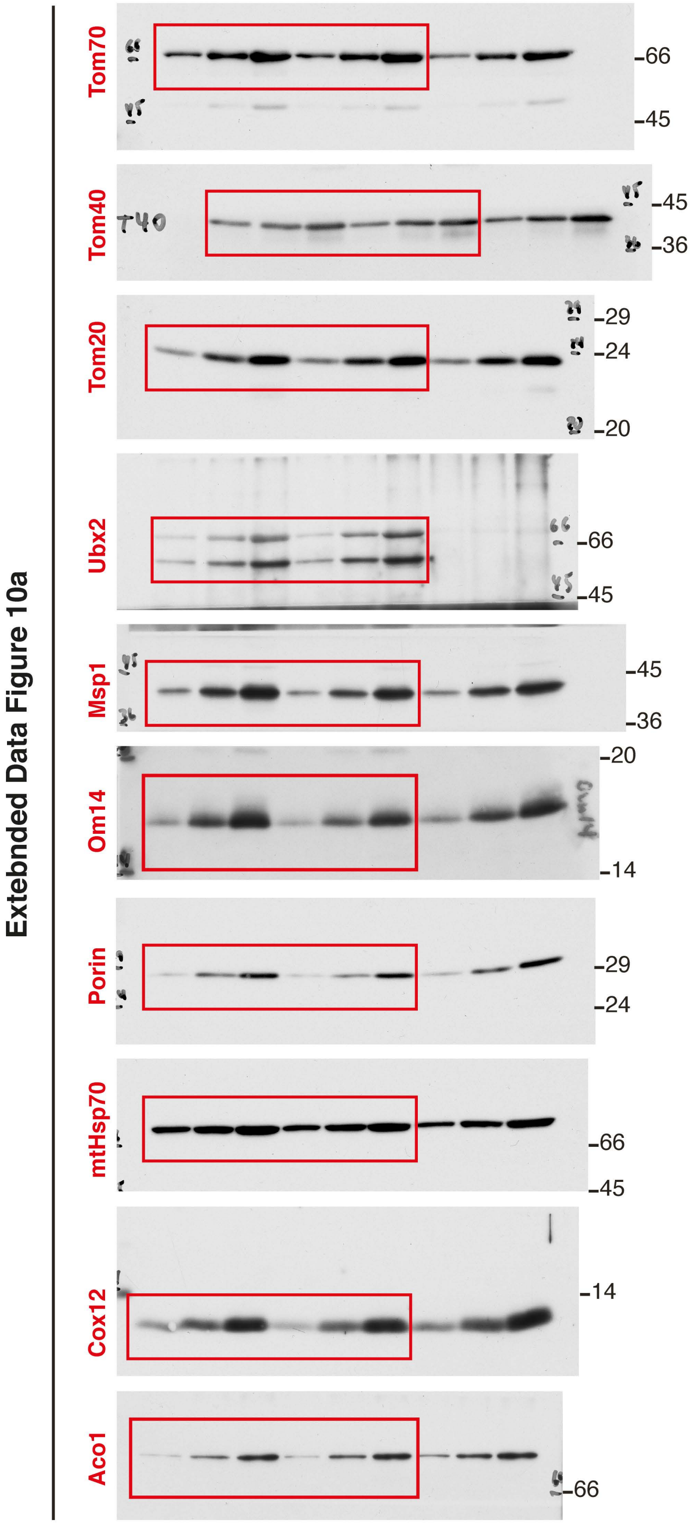
Extebnded Data Figure 10a
Tom70
66
45
Tom40
45
36
Tom20
29
24
20
Ubx2
66
45
Msp1
45
36
Om14
20
14
Porin
29
24
mtHsp70
66
45
Cox12
14
Aco1
66

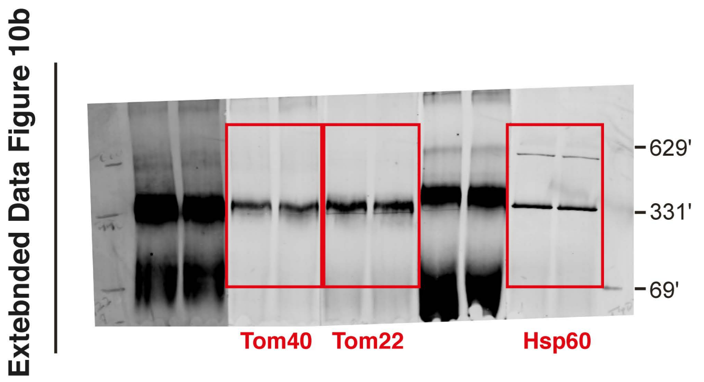
Extebnded Data Figure 10b
629'
331'
69'
Tom40
Tom22
Hsp60

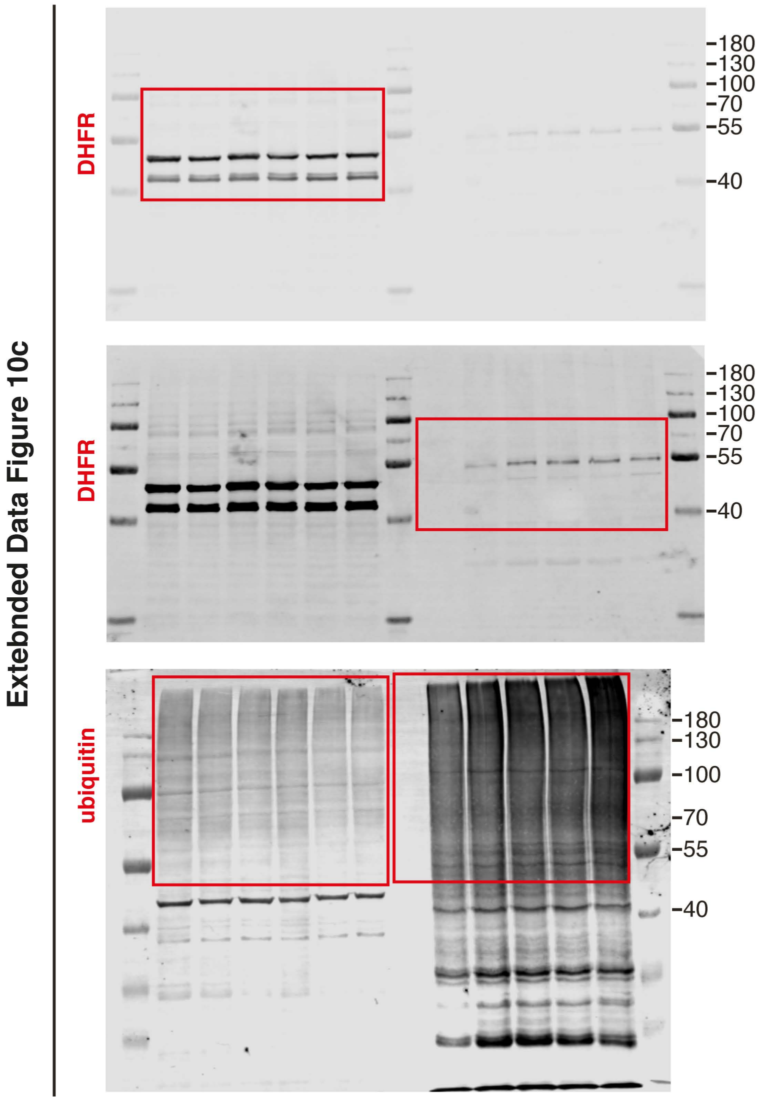
Extebnded Data Figure 10c
DHFR
180
130
100
70
55
40
DHFR
180
130
100
70
55
40
ubiquitin
180
130
100
70
55
40

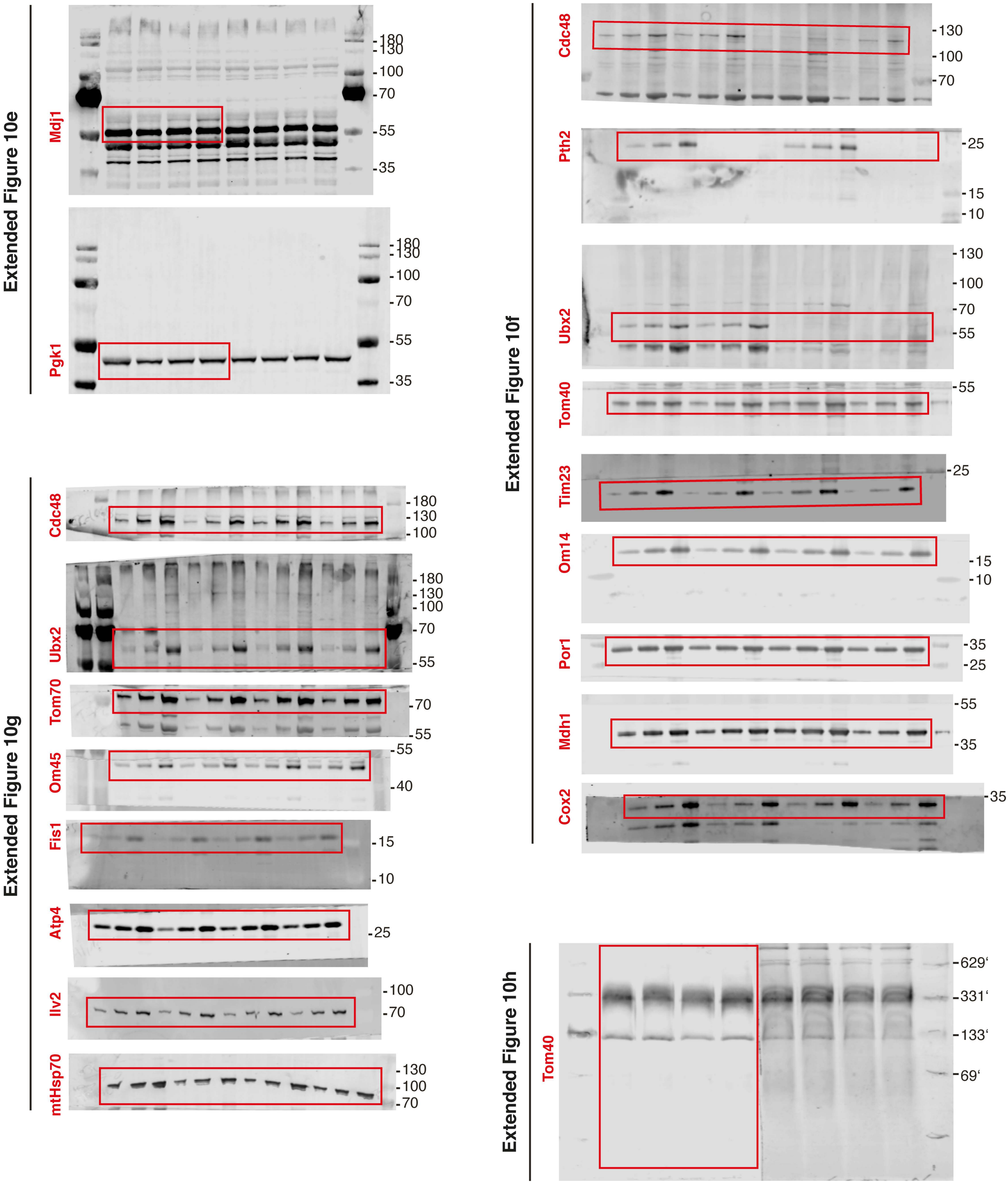

Extended Figure 10e
Mdj1
180
130
100
70
55
35
Pgk1
180
130
100
70
55
35
Extended Figure 10g
Cdc48
180
130
100
Ubx2
180
130
100
70
55
Tom70
70
55
Om45
55
40
Fis1
15
10
Atp4
25
Ilv2
100
70
mtHsp70
130
100
70
Extended Figure 10f
Cdc48
130
100
70
Pth2
25
15
10
Ubx2
130
100
70
55
Tom40
55
Tim23
25
Om14
15
10
Por1
35
25
Mdh1
55
35
Cox2
35
Extended Figure 10h
Tom40
629‘
331‘
133‘
69‘

Supplementary Table 4. Yeast strains used in this study.

Genotype	Source	Identifier
YPH499 (WT) *MATa, ura3-52, lys2-801_amber, ade2-101_ochre, trp1-Δ63, his3-Δ200, leu2Δ1*	Ref. *43*	TB1
YPH499 *cox4::COX4-10His::HIS3MX6*	Ref. *42*	NP3297
YPH499 *Phb1*$_{ProtA}$	this study	NP4063
YPH499 *Tom22*$_{His}$ *Tom40*$_{Strep}$	this study	NP4420
YPH499 *pth2::PTH2-10His::HIS3MX6*	this study	TB3
YPH499 *tom20::TOM20-10HIS-HIS3MX6*	Ref. *21*	NP3718
BY4741 (WT) *MATa, his3Δ1, leu2Δ0, met15Δ0, ura3Δ0*	EUROSCARF	TB2
BY4741 *tom40::TOM40-3HA-HIS3MX6*	Ref. *17*	TB4
BY4741 pth2*::kanMX4, tom40::TOM40-3HA-HIS3MX6*	this study	TB5
BY4741 *ubx2::kanMX4, tom40::TOM40-3HA-HIS3MX6*	Ref. *17*	TB6
BY4741 *ubx2::UBX2-3HA::HIS3MX6*	Ref. *17*	TB45
BY4741 *pth2::KanMX4*	EUROSCARF	TB8
BY4741 *pam17::hphNT1*	Ref. *17*	TB9
BY4741 *pth2::KanMX4 pam17Δ:: hphNT1*	this study	TB7
BY4741 *ubx2::KanMX4*	EUROSCARF	TB22
BY4741 *ubx2::KanMX4 pth2::HIS3MX6*	this study	TB27
BY4741 *ubp16::KanMX4*	EUROSCARF	TB21
BY4741 *ubp16::KanMX4, pth2::HIS3MX6*	this study	TB36
BY4741 *pth2::kanMX4 vms1::HIS3MX6*	this study	TB35
BY4741 *dsk2::KanMX4*	this study	TB102
BY4741 *dsk2::KanMX4, pam17::hphNT1*	this study	TB100
BY4741 *rsp5::RSP5-1-KanMX4*	EUROSCARF	TB20
BY4741 *mdm30::KanMX4*	EUROSCARF	TB101
BY4741 *mfb1::KanMX4*	EUROSCARF	TB103
BY4741 *rsp4::RSP5-1-KanMX4 ubp16::HIS3MX6*	this study	TB39
BY4741 *rsp5::RSP5-1-KanMX4 pam17::hphNT1*	this study	TB12
BY4741 *ubp16::KaMX4 pam17::hphNT1*	this study	TB13
BY4741 *pre9::kanMX4 pam17::hphNT1*	Ref. *17*	TB11
BY4741 *mdm30::KanMX4 pam17::hphNT1*	this study	TB72
BY4741 *mfb1::KanMX4 pam17::hphNT1*	this study	TB73
BY4741 *hrd1::KanMX4 pam17::hphNT1*	this study	TB71
BY4741 *doa10::KanMX4 pam17::hphNT1*	this study	TB70
BY4741 *vms1::KanMX4*	EUROSCARF	TB23
BY4741 *pth2::KanMX4 pam17Δ:: hphNT1* + pRS416	this study	TB166
BY4741 *pth2::KanMX4 pam17Δ:: hphNT1* + pRS416 pPTH2 Pth2	this study	TB167
BY4741 *pth2::KanMX4 pam17Δ:: hphNT1* + pRS416 pPTH2 Pth2 D174A	this study	TB168
BY4741 *pth2::KanMX4 pam17Δ:: hphNT1* + pRS416 pPTH2 Pth2 ΔTM (Δ aa12-32)	this study	TB169
BY4741 *pth2::kanMX4 vms1::HIS3MX6* + pRS416	this study	TB171
BY4741 *pth2::kanMX4 vms1::HIS3MX6* + pRS416 pPTH2 Pth2	this study	TB172
BY4741 *pth2::kanMX4 vms1::HIS3MX6* + pRS416 pPTH2 Pth2 D174A	this study	TB173
BY4741 *pth2::kanMX4 vms1::HIS3MX6* + pRS416 pPTH2 Pth2 ΔTM (Δ aa12-32)	this study	TB174

BY4741 *tom70::hphNT1*	this study	TB98
BY4741 *tom70::hphNT1, tom40::TOM40-3HA-HIS3MX6*	this study	TB113

Supplementary Table 5. Plasmids used in this study.

Plasmid	Source	Identifier
pDP-CYB2(1-84)-DHFR-HB(81-181) (referred to as *b*$_2$-DHFR)	Ref. *99*	pTB7
pDP-CYB2(1-84)Δ(47-65)-DHFR-HB(81-181) (referred to as *b*$_2$Δ-DHFR)	Ref. *99*	pTB8
pDP-CYB2(1-84)Δ(47-65)-DHFR(L251G, P252G)-HB(81-181) (referred to as *b*$_2$Δ-DHFR^{GGxY})	this study	pTB128
pADH His-Ubiquitin	M. Glickman, Ref. *41*	pTB3
pFA6a His3MX6	Ref. *46*	pTB2
pFA6a hphNT1	Ref. *44*	pTB6
pFA6a KanMX6	Ref. *46*	pTB1
pFA6a 3HA-His3MX6	Ref. *45*	pTB12
pRS416	Ref. *43*	pTB94
pRS416 pPTH2 Pth2	this study	pTB91
pRS416 pPTH2 Pth2 D174A	this study	pTB92
pRS416 pPTH2 Pth2 ΔTM (Δ aa12-32)	this study	pTB93

Supplementary Table 6. Antibodies used in this study.

Antibody	Source	Identifier
Rabbit polyclonal anti-Aco1	Ref. *42*	GR 947
Rabbit polyclonal anti-Atp4	Ref. *69*	GR 1970
Rabbit polyclonal anti-Cdc48	Ref. *17*	GR 5015
Rabbit polyclonal anti-Cox2	Ref. *17*	GR 1948
Rabbit polyclonal anti-Cox12	Ref. *42*	GR1937
Rabbit polyclonal anti-Cox14	Ref. *88*	GR 1544
Rabbit polyclonal anti-Fis1	Ref. *21*	GR 310
Rabbit polyclonal anti-Hsp60	Ref. *42*	170
Rabbit polyclonal anti-Ilv2	Ref. *100*	GR1010
Rabbit polyclonal anti-Mdh1	Ref. *101*	GR1088
Rabbit polyclonal anti-Mdj1	Ref. *17*	121
Rabbit polyclonal anti-Mdj2	Ref. *42*	GR 1842
Rabbit polyclonal anti-mtHsp70	Ref. *42*	GR 119
Rabbit polyclonal anti-Msp1	Ref. *17*	GR 1468
Rabbit polyclonal anti-Mss51	Ref. *42*	GR 1952
Rabbit polyclonal anti-Om14	Ref. *17*	GR 3041
Rabbit polyclonal anti-Om45	Ref. *17*	GR 1311
Rabbit polyclonal anti-Phb1	Ref. *102*	298-11
Rabbit polyclonal anti-Por1	Ref. *21*	GR 3621
Rabbit polyclonal anti-Pth2	Ref. *21*	GR 797
Rabbit polyclonal anti-Rsp5	this study	GR 5064
Rabbit polyclonal anti-Shy1	Ref. *42*	GR 1094
Rabbit polyclonal anti-Tim23	Ref. *17*	GR 133
Rabbit polyclonal anti-Tom7	Ref. *80*	GR 230
Rabbit polyclonal anti-Tom20	Ref. *69*	GR 3225
Rabbit polyclonal anti-Tom22	Ref. *69*	GR 3227
Rabbit polyclonal anti-Tom40	Ref. *69*	168
Rabbit polyclonal anti-Tom70	Ref. *69*	GR 657
Rabbit polyclonal anti-Ubp16	Ref. *21*	GR 5040
Rabbit polyclonal anti-Ubx2	Ref. *17*	GR1484
Rabbit polyclonal anti-Yta10	Ref. *6*	GR1550-3
Rabbit polyclonal anti-Yta12	this study	GR1437-3
Rabbit polyclonal anti-Pgk1	Ref. *17*	GR 753
mouse-monoclonal anti-DHFR	Santa Cruz	sc-377091
mouse-monoclonal anti-Pgk1	Invitrogen	459250
mouse-monoclonal anti-ubiquitin	Santa Cruz	sc-8017
