# Supplementary material for: Mitochondrial complexome reveals quality-control pathways of protein import
Source: Nature. 2023 Jan 25;614(7946):153–9. doi: 10.1038/s41586-022-05641-w (PMC9892010; doi:10.1038/s41586-022-05641-w)
Supplement: Supplementary file 2 — Reporting Summary [file 41586_2022_5641_MOESM2_ESM.pdf]

nature portfolio

Corresponding author(s): Nikolaus Pfanner
Bernd Fakler

Last updated by author(s): Dec 5, 2022

Reporting Summary

Nature Portfolio wishes to improve the reproducibility of the work that we publish. This form provides structure for consistency and transparency in reporting. For further information on Nature Portfolio policies, see our Editorial Policies and the Editorial Policy Checklist.

Statistics

For all statistical analyses, confirm that the following items are present in the figure legend, table legend, main text, or Methods section.

n/a	Confirmed	
☐	☒	The exact sample size (*n*) for each experimental group/condition, given as a discrete number and unit of measurement
☒	☐	A statement on whether measurements were taken from distinct samples or whether the same sample was measured repeatedly
☒	☐	The statistical test(s) used AND whether they are one- or two-sided<br>*Only common tests should be described solely by name; describe more complex techniques in the Methods section.*
☒	☐	A description of all covariates tested
☒	☐	A description of any assumptions or corrections, such as tests of normality and adjustment for multiple comparisons
☐	☒	A full description of the statistical parameters including central tendency (e.g. means) or other basic estimates (e.g. regression coefficient) AND variation (e.g. standard deviation) or associated estimates of uncertainty (e.g. confidence intervals)
☒	☐	For null hypothesis testing, the test statistic (e.g. *F*, *t*, *r*) with confidence intervals, effect sizes, degrees of freedom and *P* value noted<br>*Give P values as exact values whenever suitable.*
☒	☐	For Bayesian analysis, information on the choice of priors and Markov chain Monte Carlo settings
☒	☐	For hierarchical and complex designs, identification of the appropriate level for tests and full reporting of outcomes
☐	☒	Estimates of effect sizes (e.g. Cohen's *d*, Pearson's *r*), indicating how they were calculated

Our web collection on statistics for biologists contains articles on many of the points above.

Software and code

Policy information about availability of computer code

Data collection	LAS-3000, Amersham Imager 680 and Odyssey CLx for Western blot signals<br>QExactive HF-X mass spectrometer for acquisition of MS-data
Data analysis	Image Studio 5.2.5, ImageJ 2.1.0 and Adobe Photoshop 2021 for processing of data<br>Adobe Illustrator 2021 for figure preparation<br>msconvert v3.0.11098 for processing of primary MS data<br>MaxQuant 1.6.17 for callibration and quantification of MS data<br>Mascot 2.7 for database search (SwissProt_YEAST_20201007 + cRAP_20190304)<br>Igor Pro 9 (Wavemetrics) for data fitting and Figure preparation<br>Complexomics-mitcom 1.0 (Python package for peak detection, similarity score calculation, t-SNE visualization) available at https://doi.org/10.5281/zenodo.7355040

For manuscripts utilizing custom algorithms or software that are central to the research but not yet described in published literature, software must be made available to editors and reviewers. We strongly encourage code deposition in a community repository (e.g. GitHub). See the Nature Portfolio guidelines for submitting code & software for further information.

Data

Policy information about availability of data

All manuscripts must include a data availability statement. This statement should provide the following information, where applicable:

- Accession codes, unique identifiers, or web links for publicly available datasets
- A description of any restrictions on data availability
- For clinical datasets or third party data, please ensure that the statement adheres to our policy

The mass spectrometric (MS) data generated during this study are available via ProteomeXchange with identifier PXD029548 https://doi.org/10.6019/PXD029548. UniProtKB/Swiss-Prot (https://www.uniprot.org; SwissProt_YEAST_20201007) and Saccharomyces Genome Database (SGD, https://www.yeastgenome.org; GO term mapping 20201027) were used as resource for structural, cell biological and functional annotation of proteins. The MitCOM datasets including an interactive profile viewer are available via https://www.complexomics.org/datasets/mitcom or the CEDAR platform https://www3.cmbi.umcn.nl/cedar/browse/experiments/CRX36. All other data are available in the main figures, Extended Data and Supplementary Information, including uncropped versions of gels/blots in Supplementary Fig. 1, and Supplementary Tables 1-3 (Excel files).

Field-specific reporting

Please select the one below that is the best fit for your research. If you are not sure, read the appropriate sections before making your selection.

☒ Life sciences ☐ Behavioural & social sciences ☐ Ecological, evolutionary & environmental sciences

For a reference copy of the document with all sections, see nature.com/documents/nr-reporting-summary-flat.pdf

Life sciences study design

All studies must disclose on these points even when the disclosure is negative.

Sample size	The sample size was determined based on previous experience with specific types of experiments like the amount of mitochondria used for steady state and pulldown analysis (Martensson et al., 2019, Priesnitz et al., 2021). According to this the required amount of mitochondrial or cellular proteins was selected for each experiment. For key experiments, several runs were performed to determine the optimal sample size. The sample size or reference of sample size of each experiment is stated in the the Methods section.
Data exclusions	No data were excluded from this study. All relevant data are shown.
Replication	Representative images are shown for growth and biochemical assays/western blotting, including analysis of yeast growth (wild-type and mutants), total cell extracts, affinity purification from cell extracts, subcellular fractionation, protein steady state levels, blue native electrophoresis and affinity purification from isolated mitochondria. The findings were confirmed by independent experiments; in the main figures this applies to the following figures (minimum number of independent experiments in parentheses): 3b (2), 3d (3), 3e (2), 4b (2), 4c (2), 4d (2), 4e (2), 4f (2), 4g (2), 5a (2), 5b (3), 5c (2), 5d (3), 5e (2), 5f (3), and 5g (2). In the Extended Data figures this applies to: ED8f (3), ED8h (2), ED9b (2), ED9c (2), ED9d (2), ED9e (2), ED9f (5), ED9g (3), ED9h (2), ED10a (2), ED10b (2), ED10c (3), ED10d (2), ED10e (2), ED10f (2), ED10g (2), and ED10h (2).
Randomization	Random clones of the used yeast strains were selected for biochemical assays and mitochondrial isolations. The experiments from mitochondria were not randomized. All samples in one experiment were treated at the same time and with the same conditions.
Blinding	Blinding was not performed. The used yeast strains have to be validated before use. Thus, blinding is technically not feasible for the cell biological and biochemical assays performed. Also blinding is not required as all samples are treated in parallel.

Reporting for specific materials, systems and methods

We require information from authors about some types of materials, experimental systems and methods used in many studies. Here, indicate whether each material, system or method listed is relevant to your study. If you are not sure if a list item applies to your research, read the appropriate section before selecting a response.

Materials & experimental systems

n/a	Involved in the study	
☐	☒	Antibodies
☐	☒	Eukaryotic cell lines
☒	☐	Palaeontology and archaeology
☒	☐	Animals and other organisms
☒	☐	Human research participants
☒	☐	Clinical data
☒	☐	Dual use research of concern

Methods

n/a	Involved in the study	
☒	☐	ChIP-seq
☒	☐	Flow cytometry
☒	☐	MRI-based neuroimaging

Antibodies

Antibodies used

Antibodies against proteins from baker's yeast Saccharomyces cerevisiae were generated in rabbits using peptides (Aco1 (GR947), Atp4 (GR1970), Cdc48 (GR5015), Cox2 (GR1948), Cox12 (GR1937), Cox14 (GR1544), Fis1 (GR310), Hsp60 (170), Ilv2 (GR1010), Mdh1 (GR1088), Mdj1 (121), Mdj2 (GR1842), mtHsp70 (GR119), Msp1 (GR1468), Mss51 (GR1952), Om14 (GR3041), Om45 (GR1311), Phb1 (298-11), Por1 (GR3621), Pth2 (GR797), Rsp5 (GR5064), Shy1 (GR1094), Tim23 (GR133), Tom7 (GR230), Ubp16 (GR5040), Ubx2 (GR1484), Yta10 (GR1550), Yta12 (GR1437), Pgk1 (GR753)) or recombinant proteins (Tom20 (GR3225), Tom22 (GR3227), Tom40 (168), Tom70 (GR657). The antisera were used in 1:250-1,000 dilution. Anti-DHFR (A9; Cat. sc-377091; dilution 1:1,000) and anti-ubiquitin (P4D1; Cat. sc-8017; dilution 1:1,000) antibodies were obtained from Santa Cruz Biotechnology. Anti-Pgk1 antibody (22C5D8, Cat. 459250; dilution 1:5,000) was obtained from Invitrogen. Secondary antibodies were obtained from Dianova (HRP-conjugated goat anti-rabbit, Cart. 111-035-003) or Li-Cor (goat anti-mouse IgG, IRDye 800CW, Cat. 926-32210; goat anti-rabbit IgG, IRDye 800CW, Cat. 926-32211; goat anit-mouse IgG, IRDye 680RD, Cat. 926-68070, goat anti-rabbit IgG, IRDye 680RD, Cat. 926-68071). Secondary antibodies were used at a concentration of 1:5,000 (HRP) or 1:10,000 (IRDye).

Validation

The specificity of the antibody raised against a protein from baker´s yeast (Saccharomyces cerevisiae) was controlled by comparing total cell extracts or mitochondrial lysates from wild-type yeast cells and the corresponding deletion strain or strains expressing a tagged version of the protein of interest via SDS-PAGE and Western blotting. Absence or size shift of the signal in cellular fractions of the mutant strain confirmed the specificity of the antibody signal. References for the used antibodies are:
Rabbit polyclonal anti-Aco1, Ref. 42
Rabbit polyclonal anti-Atp4, Ref. 69
Rabbit polyclonal anti-Cdc48, Ref. 17
Rabbit polyclonal anti-Cox2, Ref. 17
Rabbit polyclonal anti-Cox12, Ref. 42
Rabbit polyclonal anti-Cox14, Ref. 88
Rabbit polyclonal anti-Fis1, Ref. 21
Rabbit polyclonal anti-Hsp60, Ref. 42
Rabbit polyclonal anti-Ilv2, Ref. 100
Rabbit polyclonal anti-Mdh1, Ref. 101
Rabbit polyclonal anti-Mdj1, Ref. 17
Rabbit polyclonal anti-Mdj2, Ref. 42
Rabbit polyclonal anti-mtHsp70, Ref. 42
Rabbit polyclonal anti-Msp1, Ref. 17
Rabbit polyclonal anti-Mss51, Ref. 42
Rabbit polyclonal anti-Om14, Ref. 17
Rabbit polyclonal anti-Om45, Ref. 17
Rabbit polyclonal anti-Phb1, Ref. 102
Rabbit polyclonal anti-Por1, Ref. 21
Rabbit polyclonal anti-Pth2, Ref. 21
Rabbit polyclonal anti-Rsp5, this study
Rabbit polyclonal anti-Shy1, Ref. 42
Rabbit polyclonal anti-Tim23, Ref. 17
Rabbit polyclonal anti-Tom7, Ref. 80
Rabbit polyclonal anti-Tom20, Ref. 69
Rabbit polyclonal anti-Tom22, Ref. 69
Rabbit polyclonal anti-Tom40, Ref. 69
Rabbit polyclonal anti-Tom70, Ref. 69
Rabbit polyclonal anti-Ubp16, Ref. 21
Rabbit polyclonal anti-Ubx2, Ref. 17
Rabbit polyclonal anti-Yta10, Ref. 6
Rabbit polyclonal anti-Yta12, this study
Rabbit polyclonal anti-Pgk1, Ref. 17
mouse-monoclonal anti-DHFR, Santa Cruz, sc-377091
mouse-monoclonal anti-Pgk1, Invitrogen, 459250
mouse-monoclonal anti-ubiquitin, Santa Cruz, sc-8017

Eukaryotic cell lines

Policy information about cell lines

Cell line source(s)

All yeast strains used in this study are described in Supplementary Table 4.
The yeast strains BY4741, pth2Δ, ubx2Δ, ubp16Δ, rsp5-1, mdm30Δ, mfb1Δ, and vms1Δ were obtained from EUROSCARF.

The yeast strains YPH499, Cox4-His, Tom20-His, Tom40-HA, ubx2Δ Tom40-HA, Ubx2-HA, pam17Δ, and pre9Δ pam17Δ have been described:

Sikorski, R. S. & Hieter, P. A system of shuttle vectors and yeast host strains designed for efficient manipulation of DNA in Saccharomyces cerevisiae. Genetics 122, 19-27 (1989).

Böttinger L. et al., A complex of Cox4 and mitochondrial Hsp70 plays an important role in the assembly of the cytochrome c oxidase. Mol. Biol. Cell 24, 2609-2619 (2013).

Mårtensson, C. U. et al. Mitochondrial protein translocation-associated degradation. Nature 569, 679-683 (2019).

Doan, K. N. et al. The mitochondrial import complex MIM functions as main translocase for α-helical outer membrane proteins. Cell Rep. 31, 107567 (2020).

The following strains were newly generated for this study: Phb1-ProtA, Tom22His Tom40-Strep, Pth2-His, pth2Δ Tom40-HA, pth2Δ pam17Δ, ubx2Δ pth2Δ, ubp16Δ pth2Δ, pth2Δ vms1Δ, dsk2Δ, dsk2Δ pam17Δ, rsp5-1 ubp16Δ, rsp5-1 pam17, ubp16 pam17, mdm30Δ pam17Δ, mfb1Δ pam17Δ, hrd1Δ pam17Δ, doa10Δ pam17Δ, pth2Δ pam17Δ, pth2Δ pam17Δ + prs416, pth2Δ pam17Δ + prs416 Pth2, pth2Δ pam17Δ + prs416, pth2Δ pam17Δ + prs416 Pth2 D174A, pth2Δ pam17Δ + prs416, pth2Δ pam17Δ + prs416 Pth2 ΔTM, pth2Δ vms1Δ + prs416, pth2Δ vms1Δ + prs416 Pth2, pth2Δ vms1Δ + prs416 Pth2 D174A and pth2Δ vms1Δ + prs416 Pth2 ΔTM, tom70Δ, tom70Δ Tom40-HA.

Authentication

Yeast strains were grown on selective media for several generations. Deletion or tagging of genes was confirmed by western blot analysis of total cell extracts using the corresponding antibodies.

Mycoplasma contamination

Contamination with Mycoplasma is not an issue in yeast cultures and was therefore not tested.

Commonly misidentified lines
(See ICLAC register)

Commonly misidentified lines were not used in this study.
